# Supplementary material for: Anthropogenic iron oxide aerosols enhance atmospheric heating
Source: Nat Commun. 2017 May 16;8:15329. doi: 10.1038/ncomms15329 (PMC5440854; doi:10.1038/ncomms15329)
Supplement: Supplementary Information — Supplementary Figures, Supplementary Tables and Supplementary References [file ncomms15329-s1.pdf]

Supplementary Table 1. Geometry and flow rate of the individual components of the aerosol sampling system used in the A-FORCE 2013W campaign.

Component	Inner diameter [mm]	Geometry	Volumetric flow rate [L min^{-1}]
Shrouded solid diffuser inlet	5.1 (tip)	See Methods section	80
1″ tube	22	1.5-m length	80
1″ bend	22	45° bend	80
3/4″ tube	16	1.5-m length	80
3/4″ bend	16	45° bend	80
1/4″ tube	4.0	2-m length	2
1/4″ bend	4.0	90° bend	2
1/8″ tube	2.0	0.3-m length	0.1
1/8″ bend	2.0	90° bend	0.1
SP2 aerosol jet	2.0 (inlet)<br>0.5 (outlet)	Abrupt Constriction	0.1

Supplementary Table 2. Wavelength-dependent complex refractive indices of BC and FeO_x.

Wavelength [nm]	BC*	FeO_x†
300	1.84 + 0.7*i*	2.18 + 0.92*i*
400	1.88 + 0.69*i*	2.41 + 0.81*i*
500	1.94 + 0.66*i*	2.5 + 0.65*i*
600	1.99 + 0.64*i*	2.56 + 0.57*i*
700	2.03 + 0.63*i*	2.56 + 0.45*i*
800	2.07 + 0.61*i*	2.48 + 0.37*i*
900	2.09 + 0.6*i*	2.40 + 0.37*i*
1000	2.12 + 0.59*i*	2.30 + 0.46*i*
1500	2.14 + 0.65*i*	2.58 + 1.10*i*
2000	2.17 + 0.75*i*	2.93 + 1.30*i*
2500	2.21 + 0.86*i*	3.00 + 1.40*i*

* Refractive index of soot reported by Bergstrom [1].

† Refractive index of magnetite reported by Huffman and Stapp [2].

Supplementary Table 3. Input parameters for radiate transfer calculations.

Parameter's name	Input value
Radiative transfer solver	sdisort, 8-streams, delta-m method
Gas absorption parameterization	LOWTRAN/SBDART parameterization
Wavelength range	250–2500 nm
Aerosol profile	8 km-TOA: No aerosols<br><br>0–1 km, 1–2 km, 2–4 km, 4–6 km, 6–8 km: Homogeneous layer with<br>SSA = 0.85,<br>Asymmetry factor = 0.7,<br>Henyey-Greenstein phase function,<br>Absorption coefficient was computed from the BC and FeO_x data.
Atmospheric profile	"US-standard atmosphere" with<br>CO_2 mixing ratio = 400 ppmv
Latitude and longitude	36.0°N, 125.0°E
Solar zenith angle	Effective solar zenith angle<br>averaged for<br>local noon − 6 h<br>~ local noon + 6 h<br>on March 04, 2013
Surface albedo	IGBP surface type 17<br>(ocean water)

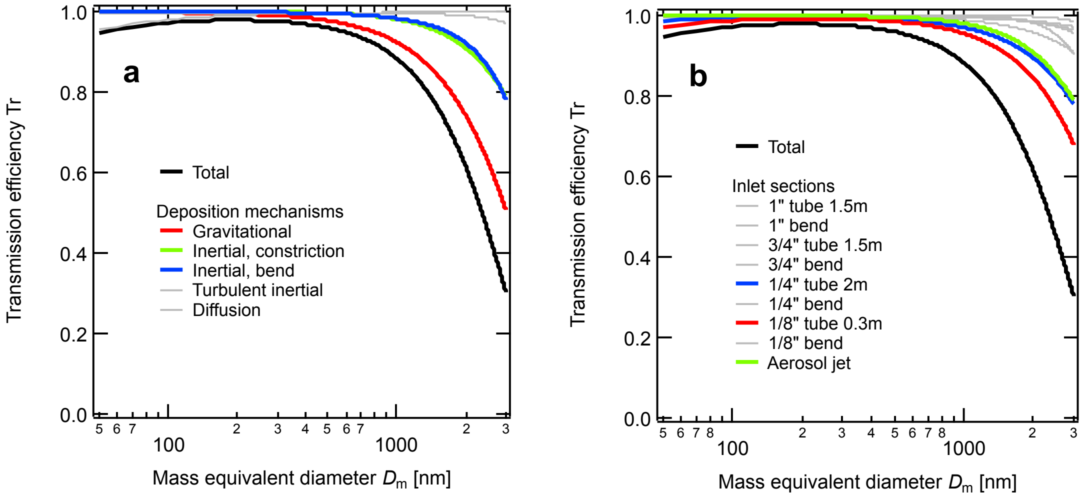

Supplementary Figure 1. Transmission efficiency curves Tr(D_m) of FeO_x particles for the aerosol sampling system calculated assuming the observation conditions around an altitude of 1 km. The parameters of the aerosol sampling system used for the Tr(D_m) calculations are listed in Supplementary Table 1. The black lines in each panel represent the total Tr(D_m). Panel (a) shows the Tr(D_m) curves for the individual deposition mechanisms, and panel (b) shows those of the individual components comprising the aerosol sampling system. The Tr(D_m) curves calculated assuming the observation conditions at other altitudes were similar to these results.

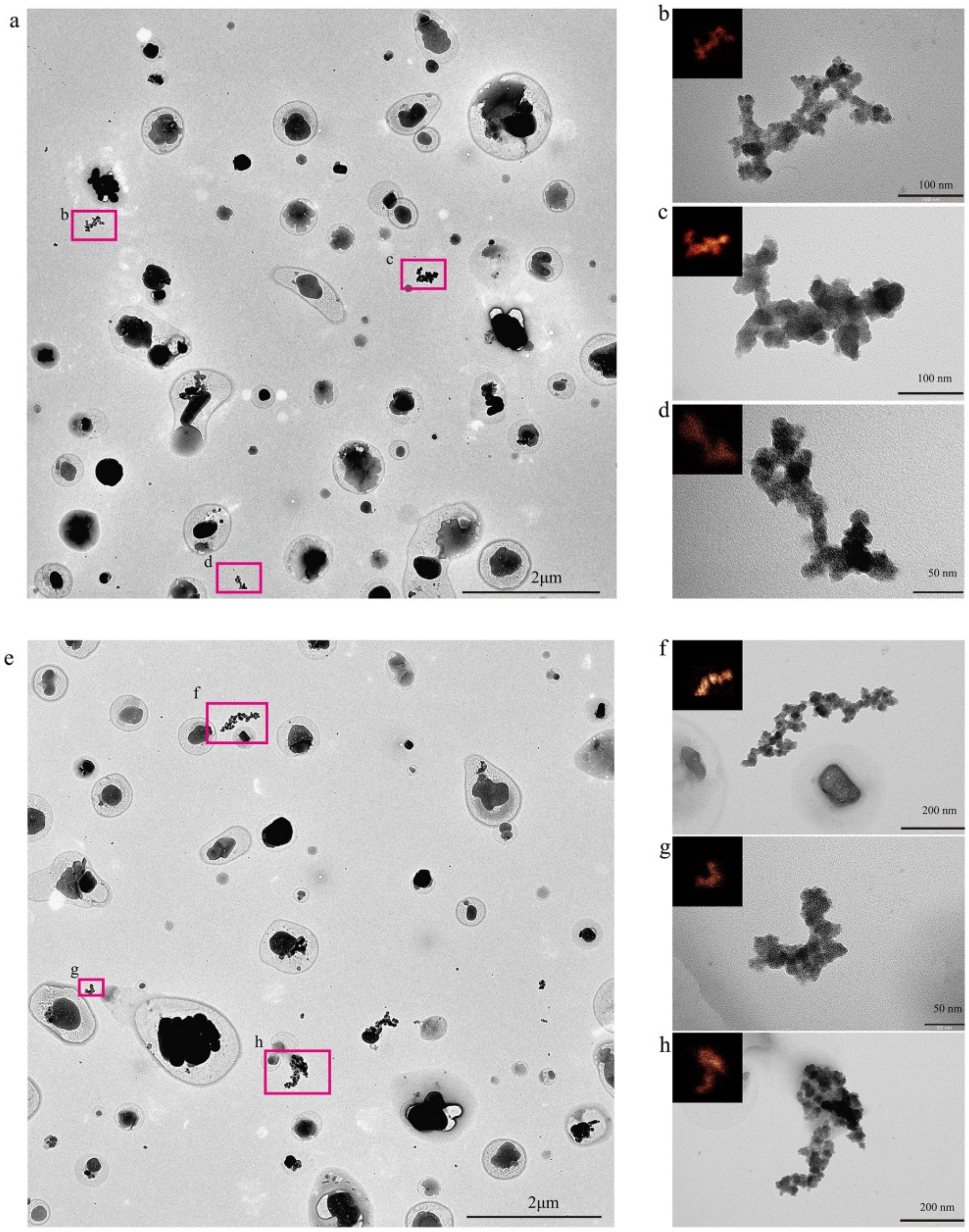

Supplementary Figure 2. Transmission electron microscopy (TEM) images of aggregated FeO_x nanoparticles found in dry PBL air. The sample was collected by an aerosol-impactor sampler onboard the aircraft from 12:53 to 13:05 on March 7, 2013 (local time) during A-FORCE 2013W campaign (i.e., Sample number 3 in Table 1). (a) and (e): TEM images of aerosol particles collected on the substrate. Red squares with alphabet indicate the aggregated FeO_x nanoparticles shown in (b)-(d) and (f)-(h). Upper left insets in (b)-(d) and (f)-(h) are distributions of Fe.

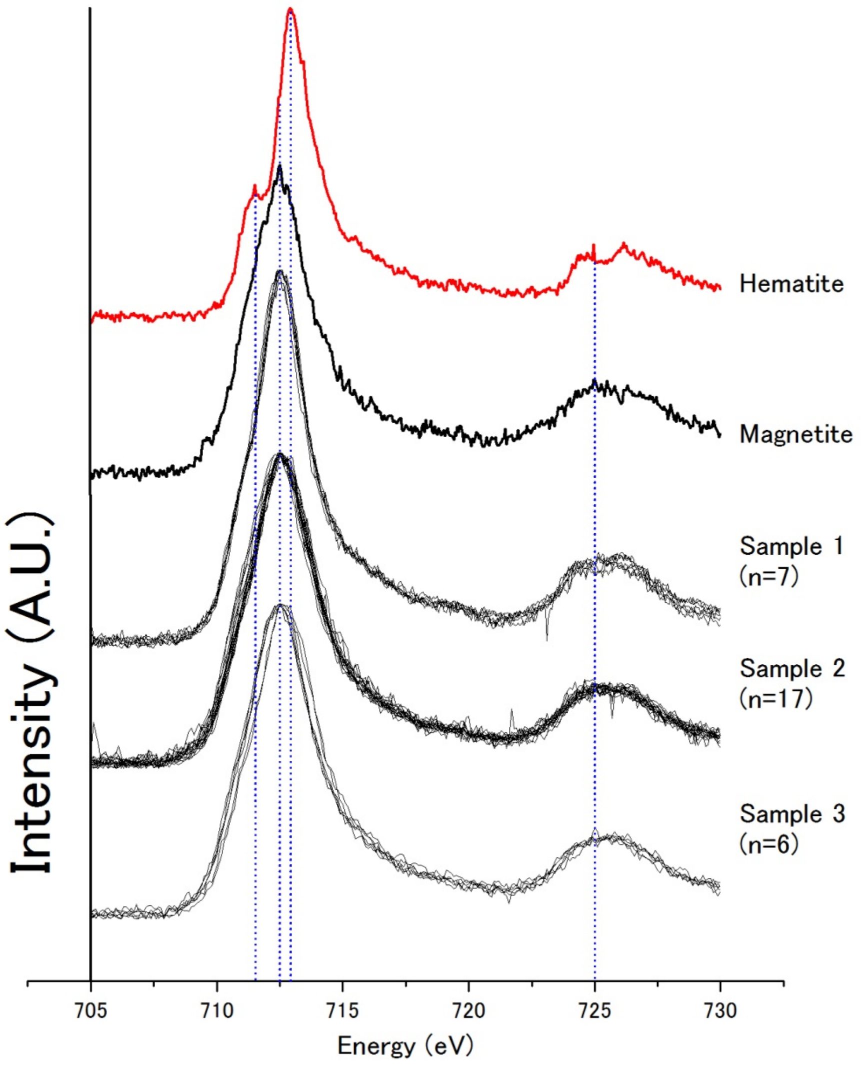

Supplementary Figure 3. TEM and electron energy loss spectroscopy (EELS) spectra of laboratory and ambient FeO_x particles. Hematite and magnetite indicate the results for the laboratory FeO_x particles used in our SP2 characterizations [3]. Ambient FeO_x indicates the results for aggregated FeO_x nanoparticles found in the aerosol-impactor samples collected in dry PBL air. We used the three samples listed in Table 1: Sample 1, 14:35 to 14:47 on Mar. 4; Sample 2, 11:53 to 12:05 on Mar. 7; and Sample 3, 12:53 to 13:05 on Mar. 8. The vertical dotted lines indicate peak positions. The EELS spectra of the ambient particles are similar to that of magnetite, and no hematite particles were observed among the measured FeO_x particles. These EELS peaks indicate Fe^{2+} and Fe^{3+} along with the L_3 and L_2 edges [4, 5].

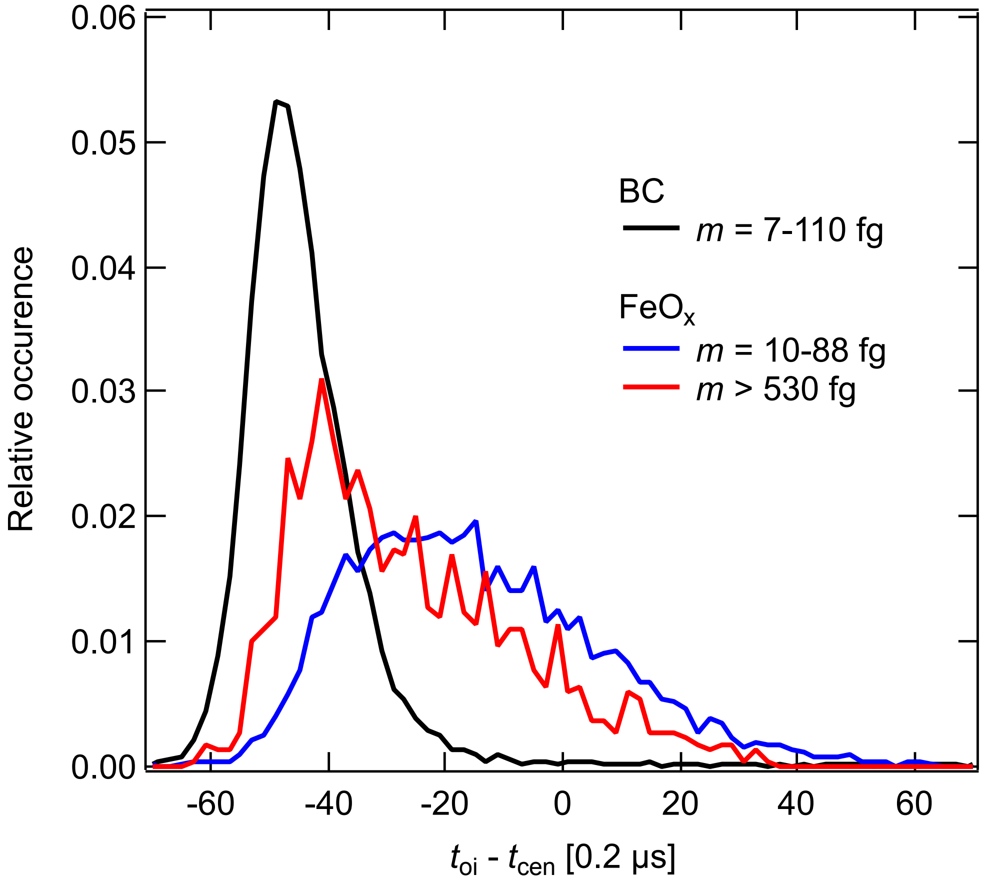

Supplementary Figure 4. Timing of the onset of incandescence (t_{oi}) relative to the timing at the center of the Gaussian beam (t_{cen}) [3] for incandescing particles in dry PBL air. The distribution is shown for a selected range of particle masses (m).

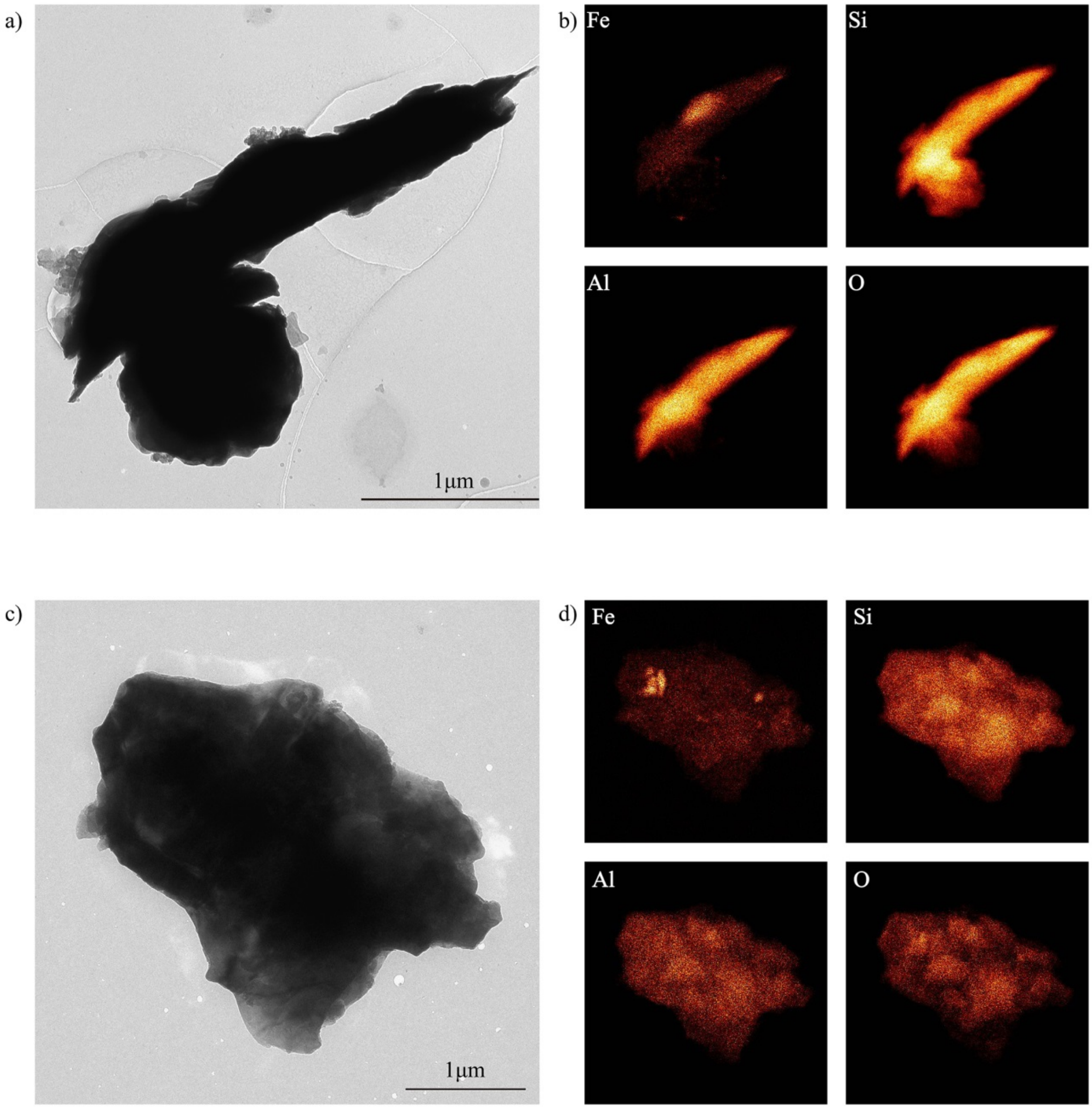

Supplementary Figure 5. Transmission electron microscopy (TEM) images of two Fe-bearing mineral dust particles found in the highest altitude 6-8 km. The sample was collected by an aerosol-impactor sampler onboard the aircraft from 11:40 to 11:52 on March 2, 2013 (local time) during A-FORCE 2013W campaign. (a) and (c): TEM images of mineral dust particles. Images in (b) and (d) indicate element distributions for Fe, Si, Al, and O. They mainly consist of Si and Al, suggesting that they are mostly aluminosilicate dust particles. They also contain Fe-rich parts, which may be detectable by the SP2.

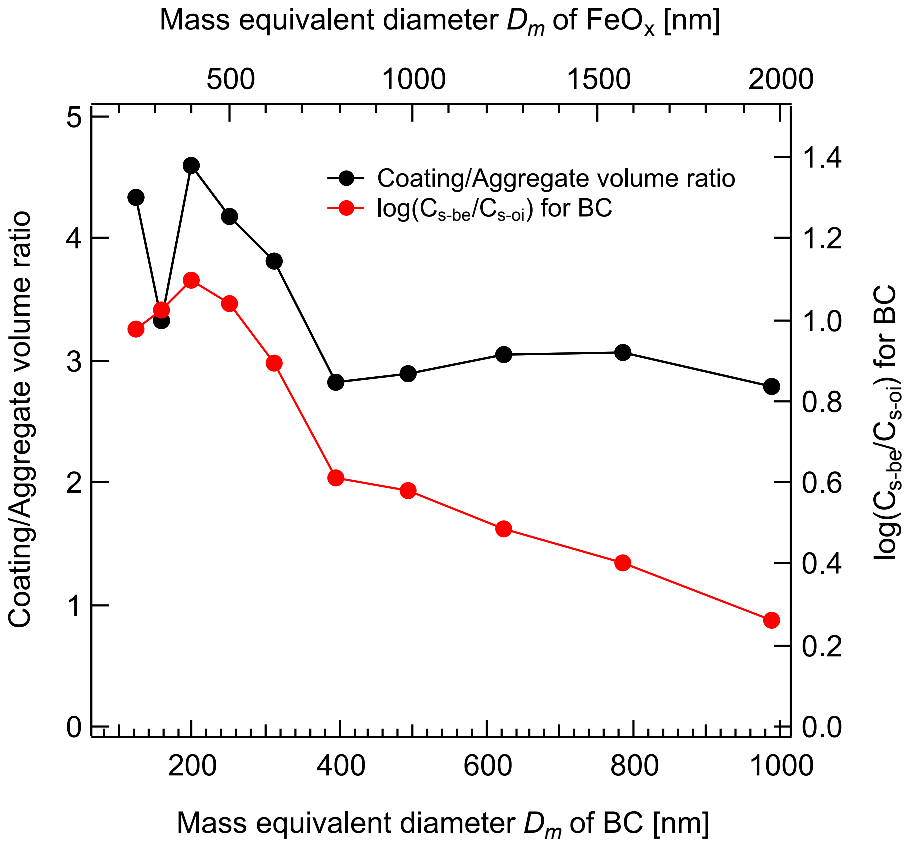

Supplementary Figure 6. The coating/aggregate volume ratio as a function of aggregate mass equivalent diameter D_m prescribed for coated model particles. The simulated $\log(C_{s\text{-}be}/C_{s\text{-}oi})$ values for model BC-containing particles are also shown. Here, we assume that the particle's scattering cross sections with and without coating are equal to $C_{s\text{-}be}$ and $C_{s\text{-}oi}$, respectively.

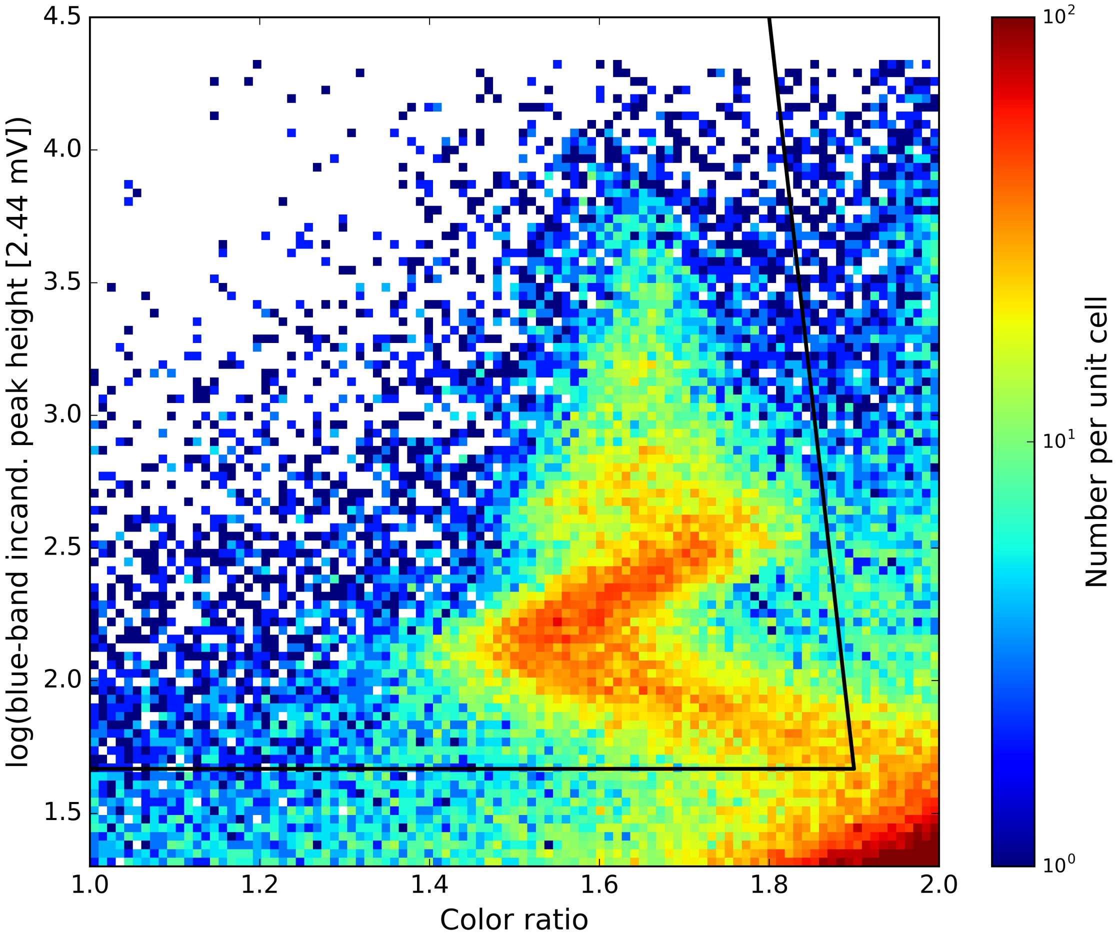

Supplementary Figure 7. Scatterplot of the peak amplitude of the blue-band incandescence peak and the color ratio for all incandescing particles detected during the A-FORCE 2013 W campaign. Color ratios greater than 2.0 are omitted from the figure. The boundary lines for discriminating FeO_x from BC are also shown (black lines).

Supplementary References

1. Bergstrom, R.W. Predictions of the spectral absorption and extinction coefficients of an urban air pollution aerosol model. *Atmospheric Environment* 6, 247–258 (1972).
2. Huffman D. R. & Stapp, J. L. *Interstellar Dust and Related Topics,* J. M. Greenberg and H. C. Van de Hulst, eds. Reidel, Boston (1973), pp. 297–301.
3. Yoshida, A. *et al.* Detection of light-absorbing iron oxide particles using a modified single-particle soot photometer. *Aerosol Science and Technology* 50, 1–4 (2016).
4. Maher, B. *et al*. Magnetite pollution nanoparticles in the human brain. *Proceedings of the National Academy of Sciences* 113, 10797–10801 (2016).
5. Garvie, L. A. J. & Buseck, P. R. Ratios of ferrous to ferric iron from nanometre-sized areas in minerals. *Nature* 396, 667–670 (1998).
